# Interaction of HnRNP F with the guanine-rich segments in viral antigenomic RNA enhances porcine reproductive and respiratory syndrome virus-2 replication

**DOI:** 10.1186/s12985-022-01811-4

**Published:** 2022-05-15

**Authors:** Aiguo Zhang, Yanting Sun, Huiyuan Jing, Jie Liu, Erzhen Duan, Wenting Ke, Ran Tao, Yang Li, Jinhe Wang, Sufang Cao, Pandeng Zhao, Haihua Wang, Yan Zhang

**Affiliations:** 1 Key Laboratory of Veterinary Biological Products, College of Veterinary Medicine, Henan University of Animal Husbandry and Economy, Zhengzhou, 450046 China; 2 College of Animal and Veterinary Sciences, Southwest Minzu University, Chengdu, 610041 China; 3 College of Biological Engineering, Henan University of Technology, Zhengzhou, 450001 China; 4 State Key Laboratory of Agricultural Microbiology, College of Veterinary Medicine, Huazhong Agricultural University, Wuhan, 430070 China

**Keywords:** Porcine reproductive and respiratory syndrome virus (PRRSV), HnRNP F, Guanine-rich segments (GRS), Nucleocapsid (N) protein, Replication

## Abstract

**Background:**

Heterogeneous nuclear ribonucleoprotein (HnRNP) F is a member of HnRNP family proteins that participate in splicing of cellular newly synthesized mRNAs by specifically recognizing tandem guanine-tracts (G-tracts) RNA sequences. Whether HnRNP F could recognize viral-derived tandem G-tracts and affect virus replication remain poorly defined.

**Methods:**

The effect of HnRNP F on porcine reproductive and respiratory syndrome virus (PRRSV) propagation was evaluated by real-time PCR, western blotting, and plaque-forming unit assay. The association between HnRNP F and PRRSV guanine-rich segments (GRS) were analyzed by RNA pulldown and RNA immunoprecipitation. The expression pattern of HnRNP F was investigated by western blotting and nuclear and cytoplasmic fractionation.

**Results:**

Knockdown of endogenous HnRNP F effectively blocks the synthesis of viral RNA and nucleocapsid (N) protein. Conversely, overexpression of porcine HnRNP F has the opposite effect. Moreover, RNA pulldown and RNA immunoprecipitation assays reveal that the qRMM1 and qRRM2 domains of HnRNP F recognize the GRS in PRRSV antigenomic RNA. Finally, HnRNP F is redistributed into the cytoplasm and forms a complex with guanine-quadruplex (G4) helicase DHX36 during PRRSV infection.

**Conclusions:**

These findings elucidate the potential functions of HnRNP F in regulating the proliferation of PRRSV and contribute to a better molecular understanding of host-PRRSV interactions.

## Background

Porcine reproductive and respiratory syndrome (PRRS), caused by the PRRS virus (PRRSV), is a significant threat to the global pig industry [1]. PRRSV is a member of the *Arteriviridae* family within the order *Nidovirales.* PRRSV can be grouped into two genotypes, namely European (PRRSV-11) and North American (PRRSV-2), represented by Lelystad virus and VR-2332 as the prototype virus for each genotype, respectively [2]. In China, PRRSV was first reported and isolated in 1996 (CH-1a strain). In 2006, highly pathogenic PRRSV (HP-PRRSV) strains emerged in mainland China and certain strains, such as WH3, JXA1, JXwn06, HuN4, and TJ, became the predominant strains [3]. Subsequently, lineage 3 (QYYZ), NADC30-like (WUH6 and CHsx1401), and NADC34-like PRRSV were reported in recent years [4, 5]. Antibodies isolated from pigs exposed to diverse PRRSV strains were mainly found to be GP5-specific and were determined to neutralize homologous but not heterologous PRRSV strains [6]. The extensive genetic variation, mutation, and recombination make the prevention and control of PRRSV challenging [7]. Since no specific anti-viral drug has been available to treat PRRSV infection, understanding the molecular mechanisms of virus-host interactions may help identify new therapeutic strategies to combat PRRS.

The PRRSV genome is a positive-sense, single-stranded RNA that encodes ten open reading frames (ORF): ORF1a, ORF1ab, ORF2a, ORF2b, and ORFs 3–7 [8]. Like most single-strand positive RNA viruses, PRRSV genome RNA can be directly translated into two large polyprotein precursors, which are further processed post-translationally to generate more than 14 non-structural proteins (Nsps), including Nsp9, the RNA-dependent RNA polymerase (RdRp). Upon production of antigenomic RNA (minus-strand) by the Nsp9, both positive-sense genomic RNA and plus-strand sub-genomic RNAs can generated, which are subsequently translated for structural proteins [9]. The RNA comprising the PRRSV genome is packaged by nucleocapsid (N) proteins encoded by sub-genomic RNA. As the most abundant viral protein expressed during infection, N protein contains a nuclear localization signal and is distributed in the nucleus and cytoplasm of infected cells [10]. Previous studies have identified multiple cellular proteins that regulate PRRSV replication through interacting with N protein, suggesting that N protein is essential for viral replication [11–16].

Members of the heterogeneous nuclear ribonucleoproteins (HnRNPs) comprise a family of multi-purpose RNA binding proteins that are linked to numerous pathways of cellular nucleic acid metabolism both under normal and pathological conditions [17]. The HnRNP F/H proteins constitute a subclass of HnRNPs that plays overlap but context-dependent biological roles [18]. It has been reported that HnRNP F, which possesses three quasi-RNA recognition motifs (qRRM), regulates mRNA metabolism by binding to guanine-rich segments (GRS) with interspersed U/A RNA in the target exons and adjacent introns [19]. Notably, these sequences have the potential to fold into stable secondary structures known as RNA guanine-quadruplex (G4) [20]. Previous studies on compounds targeting viral GRS have shown that the stabilization of G4 structures leads to anti-viral activity [21–23]. Understanding the mechanisms of how viruses process the extremely stable structure to establish successful infection may provide new insights into drug development.

SILAC coupled to affinity purification using Flag-traps and LC–MS/MS previously identified HnRNP F, known to participate in cellular GRS recognition, as a potential N protein-interacting candidate [24]. However, whether swine HnRNP F can associate with PRRSV RNA and impact viral replication remain entirely unknown. This study addresses the functional significance of porcine HnRNP F during PRRSV replication, and demonstrates that HnRNP F associates with the GRS in PRRSV negative-sense RNA to enhance PRRSV proliferation. These data provide functional insight into PRRSV GRS and suggest novel mechanisms regulating PRRSV replication by HnRNP F.

## Materials and methods

### Cells and viruses

Cells were purchased from Procell life science & technology Co., Ltd (Wuhan, China) and authenticated by the supplier. MARC-145 cells, an epithelial-derived African green monkey kidney cell line, and human embryonic kidney (HEK) 293 T cells were maintained in Dulbecco's Modified Eagle Medium (DMEM) (Solarbio, Beijing, China) containing 100 units/mL penicillin and 100 mg/mL streptomycin supplemented with 10% heat-inactivated fetal bovine serum (Solarbio), in a 5% CO_2_ atmosphere. Porcine alveolar macrophages (PAMs) were cultured and maintained in RPMI-1640 (Solarbio). All cells used are regularly tested and are mycoplasma-free. The prototype PRRSV VR2332 (GenBank no. U87392.3) of the North American genotype kept in our laboratory was used for all subsequent experiments in this study. PRRSV was amplified and titrated in MARC-145 cells and stored at − 80 °C. UV-inactivated PRRSV was generated by irradiating the virus with short-wave UV light (254 nm) for 1 h. The loss of infectivity was confirmed by the inability of the UV-exposed virus to produce a cytopathic effect on the monolayer of MARC-145 cells.

### Plasmid constructions and mutagenesis

Mammalian expression plasmids for HA-tagged N protein pCAGGS-HA-N (full sequence and specific mutations) have been previously described [25]. Flag-tagged HnRNP F expression vectors were generated by amplifying full-length porcine HnRNP F (GenBank accession number XM_005671001.3) by reverse transcription polymerase chain reaction, with primers (TsingKe) containing restriction sites for *Bam*H I or *Xho* I (Takara, Dalian, China) to facilitate directional cloning into the pCMV-Tag2B vector. Flag-tagged deletion mutants of HnRNP F lacking the qRRM1 (amino acid residues 1–85), qRRM2 domain (amino acid residues 111–188), and qRRM3 domain (amino acid residues 289–414) were subcloned into the pCMV-Tag2B vector using standard molecular biology techniques. Primers used in cloning and mutations are listed in Table 1. All expression plasmids were sequenced to confirm the correct tandem in-frame insertion of individual genes.

**Table 1:** The sequences of primers used for construction of porcine HnRNP F (GenBank: XM_005671001.3) mutants plasmids

Name	Primer Sequence (5'-3')
HnRNP F-F	TTT*GGATCC*ATGATGTTGGGCCCTGAGGGAGGTGAAG
HnRNP F-R	TTT*CTCGAG*CTAGTCATATCCACCCATGCTGTTCTGG
ΔqRRM1-F	TTT*GGATCC*TTCAAGTCCCACAGAACCGAGATGGAT
ΔqRRM2-F	GACACTGCCAATGATGAAGAGGTTAGGTCATACTC
ΔqRRM2-R	TGACCTAACCTCTTCATCATTGGCAGTGTCTGCAC
ΔqRRM3-R	TTT *CTCGAG*TTATCCAGTGGTGCTCTGGACAGTAAACTCG
qRRM3-F	TTT*GGATCC*GAAGAGGTTAGGTCATACTCAGATCC

The restriction enzymes *Bam*H I and *Xho* I cutting sites used for cloning are underlined in italics

### Transfect and siRNA-mediated knockdown assay

Cells were transfected with indicated expression plasmids (1.0 μg/mL) twice over a 48-h period by using Lipofectamine 3000 reagent (Invitrogen). Where necessary, an empty vector was used to maintain equal amounts of DNA among wells. The siRNAs (target sequence, 5'-GCAACACAGAUACAUAGAATT-3') against swine HnRNP F (Tsingke) or the negative control siRNA (5'-UUCUCCGAACGUGUCACGUTT-3') were transfected by using Lipofectamine RNAiMAX (Invitrogen) or LipoRNAi™ (Beyotime). Briefly, a total of 300,000 PAMs plated in 6-well plates were transfected with 30 nM control or si-HnRNP F twice over a 48-h period and then infected with PRRSV. Cell lysates were prepared for western blotting to examine the levels of HnRNP F.

### RNA isolation and real-time PCR

Total RNA from cells was extracted using Trizol reagent (Invitrogen). One microgram of purified RNA was reverse transcribed to cDNA using HIFIScript gDNA removal cDNA synthesis kit (CWbiotech, China). Real-time PCR was performed with Magic SYBR Mixture reagent (CWbiotech), using primers listed in Table 2. The relative abundance of HnRNP F transcripts was calculated by the 2^−∆∆Ct^ models. Absolute quantitative N gene RNA levels were calculated as previously described [26]. Dilutions of the pCAGGS-HA-N plasmid were used to generate standard curves. Samples were assayed three times and normalized to GAPDH or β-actin. The reaction proceeded at 95 °C for 30 s, followed by 40 cycles at 95 °C for 5 s and 60 °C for 30 s.

**Table 2:** The sequences of primers used for real-time PCR

Primer names	GenBank	Sequence (5’–3’)
*HnRNP F-F*	(XM_005671001.3)	GGTGTTCAAGTCCCACAGAA
*HnRNP F-R*		CCATTTGGCACGATTTCC
*β-actin-F*	(XM_021086047.1)	TGAGAACAGCTGCATCCACTT
*β-actin-R*		CGAAGGCAGCTCGGAGTT
*Gapdh-F*	(NM_001195426.1)	TCATGACCACAGTCCATGCC
*Gapdh-R*		GGATGACCTTGCCCACAGCC
*total vRNA-F*	(U87392.3)	AAACCAGTCCAGAGGCAA
*total vRNA-R*		CGGCAAACTAAACTCCACA

### Western blotting and Co-IP assays

Cells were lysed by immunoprecipitation lysis buffer (RIPA) (Boster, Wuhan) after transfection or stimulation at the time points indicated. Soluble proteins were separated by 8%-12.5% sodium dodecyl sulfate polyacrylamide gel electrophoresis (SDS-PAGE) (Boster) and transferred to PVDF membranes (Millipore, Billerica, MA). Run for 2–3 h at 80 V on ice. After blocking with 5% skim milk powder in TBST (Solarbio), membranes were incubated with anti-HnRNP F (Protein tech, Wuhan, China), anti-N protein (Zoonogen, Beijing, China), anti-Flag, and anti-HA primary antibody (Protein tech) for 4 h, followed by washing and incubation with horseradish peroxidase conjugated goat anti-rabbit or goat anti-mouse IgG light (or heavy) chain secondary antibody (Abbkine Science, USA) for 2 h. After three washes with TBST, proteins signal was recorded digitally using enhanced chemiluminescence reagents (Boster) by a Minichemi 610 system (Sagecreation, Beijing, China). Densitometry quantification of protein bands of interest was performed using ImageJ software.

For Co-IP experiments, HEK293T cells were transfected with various plasmid mixtures. PAMs or MARC-145 cells were infected with PRRSV, as described in the figure legends. Cells were collected and lysed by RIPA buffer (containing RNase and DNase when indicated) and subjected to immunoprecipitation by incubation with indicated antibodies overnight at 4 °C. The immunocomplexes were captured by protein A + G beads, washed five times with RIPA, resuspended in SDS-PAGE protein loading buffer (Boster), and separated by SDS-PAGE. Western blotting was performed using the indicated antibodies.

### Plaque-forming unit (PFU) assay

Virus titers were determined in MARC-145 cells. Briefly, serial tenfold dilutions of the virus were made in serum-free medium, and 0.1 mL of each dilution was added per well to a monolayer of MARC-145 cells in a 6-well plate. After 1 h of absorption at 37 °C, the supernatants were discarded, and the cells were washed with PBS. The cell monolayer was then overlaid with 2 × DMEM (Solarbio) containing 4% FBS mixed with an equal volume of 2% low-melting-point agarose (Solarbio). The plate was then inverted and incubated at 37 °C for 3–4 days. The resulting plaques were stained with 0.1% neutral red (Solarbio). Viral titers were determined as PFU/mL.

### RNA pulldown

The in vitro synthetic single-strand RNA of PRRSV GRS sequence (5′-GGGUGGGGGCGUGGGGGUCGGGUCG-3′) and the sequence with mutation (5′- GAGUGGAAGCGUGGAAGUCGAGUCG-3′) were purchased from Tsingke. Biotin-RNA pulldown assay was performed as follows. First, 10 μg biotin-GRS RNA was heated to 95 °C for 5 min in 10 mM Tris–HCl (pH 7.0) buffer containing 2 M KCl and cooled down at room temperature and then incubated with streptavidin magnetic beads (Solarbio) (20 μL) at 4 °C for 2 h. Next, 4 × 10^7^ cells were washed with PBS (Solarbio) and were gently resuspended in RIPA buffer. The solution was centrifuged at 12, 000 rpm for 10 min. 100 μL of the supernatant was kept for detection and the rest were incubated with biotin-G4-streptavidin-agarose at 4 °C overnight. The precipitated samples were washed 5 times to remove unbound proteins and then resuspended in 30 μL of elution buffer (50 mM Tris pH 8.0, 1% SDS), and boiled for 10 min. After centrifugation, the supernatant was collected for SDS-PAGE and western blotting.

### RNA immunoprecipitation (RIP) analysis

MARC-145 cells were infected with PRRSV at an MOI of 1. At 24 hpi, the cells were trypsinized, collected in PBS, and cross-linked with 1–5% formaldehyde by shaking slowly on a roller for 10–15 min at room temperature, and then glycine was added to stop the cross-linking reaction. After 10 min incubation, the cells were washed twice with ice-cold PBS and lysed in ice-cold RIPA. Immunoprecipitation was performed by incubating the cell lysates at 4˚C on a roller overnight with anti-HnRNP F or rabbit IgG. Protein A + G agarose beads were added and incubated for another 1 h. Binding was performed for 90 min in rotation and beads were washed six times with ice-cold RIPA. Cross-linking was reverted by beads resuspension in NT2 buffer (50 mM Tris–HCl [pH 7.4], 150 mmol NaCl, 1 mmol MgCl2, and 0.05% Nonidet P-40) at 42˚C for 1 h and then 65˚C for 1.5 h. Finally, the RNA was extracted. The GRS containing region of PRRSV was amplified by PCR using the following primer set: 5′-TAACATAGATGCCGAGGGC-3′ (forward) and 5′-AAGCCATTGAGACCAGAGTC-3′ (reverse). Lysates (input) were subjected to reverse transcription polymerase chain reaction (RT-PCR) in parallel. The PCR products were detected by 1.2–2% agarose gel electrophoresis.

### Nuclear and cytoplasmic fractionation

The Nuclear and cytoplasmic fraction was extracted from cells using a cytoplasmic and nuclear protein extraction kit (Aidlab Biotechnologies, Beijing, China) according to the manufacturer's instructions. Briefly, cells were infected with PRRSV or transfected with pCAGGS-HA-N. A total of 2 × 10^6^ cells were resuspended in 200 μL cytoplasmic extraction reagent A, vortexed for 15 s, and incubated for 10–15 min on ice before addition of 11 μL cytoplasmic extraction reagent B, vortexed for 5 s, and incubated for 1 min on ice. The lysates were centrifuged at 4 °C for 5 min at 12,000 rpm, and the supernatants were collected as the cytoplasmic protein. The nuclear pellet was resuspended in 50 μL nuclear extraction reagent and vortexed for 30 s. This step was repeated every 10 min four times. The nuclear pellet was centrifuged at 4 °C for 10 min at 12,000 rpm, and the supernatant was collected as a nuclear protein. The cytoplasmic and nuclear fractions were stored at − 80 °C prior to western blotting analysis.

### Confocal microscopy analysis

PAMs seeded on coverslips in 24-well plates were infected with PRRSV (MOI = 0.1). At 24 hpi, the cells were fixed with 4% paraformaldehyde for 15 min, and permeabilized with 0.1% Triton-X-100 for 15 min at room temperature. The cells were then incubated with anti-HnRNP F rabbit polyclonal antibody, followed by PRRSV N protein monoclonal antibody for 1 h. After PBS washing, the cells were stained with FITC-conjugated goat anti-rabbit and Cy3-conjugated goat anti-mouse secondary antibodies. Nuclei were stained with DAPI. Fluorescent images were acquired with a confocal laser scanning microscope (Olympus Fluoview, Japan).

### Statistical analysis

Student′s *t*-test was used to compare between two groups or one-way analysis of variance (ANOVA) for more than two groups (*P-value < 0.05 and ** *P*-value < 0.01).

## Results

### Knockdown of endogenous HnRNP F via a siRNA inhibits PRRSV multiplication

**Fig. 1:**
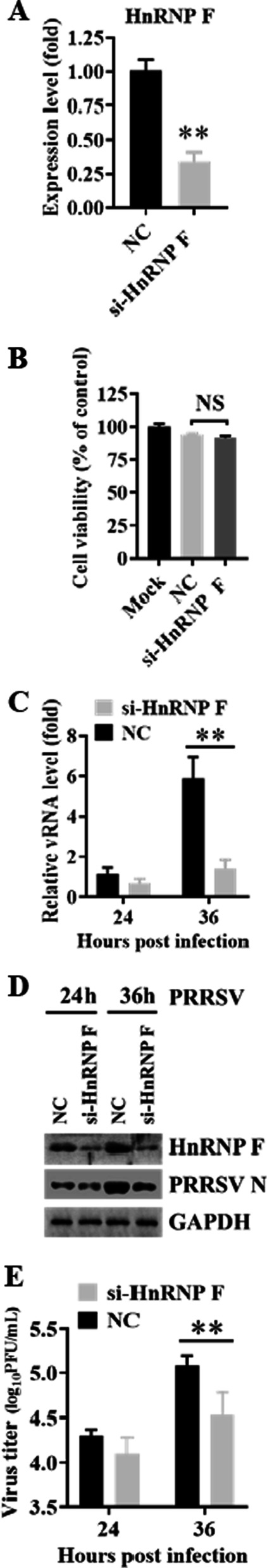
HnRNP F knockdown impairs PRRSV replication. **a** Endogenous HnRNP F mRNA levels were examined by real-time PCR in negative control-siRNA (NC) or si-HnRNP F treated PAMs to confirm the knockdown efficiency. **b** The toxicity of HnRNP F depletion toward PAMs after transfection was tested using an Enhanced Cell Counting Kit-8. **c**–**e** PAMs were transfected with the indicated siRNAs, and 24 h later were infected with PRRSV (MOI = 0.1). The cells were collected at 24–36 hpi to determine total viral RNA levels by real-time PCR (**c**), N protein expression levels by western blotting assays (**d**), and viral titers in supernatants by plaque assay (**e**), respectively

We first evaluated how depletion of endogenous HnRNP F modified the multiplication of PRRSV in PAMs, the natural host cell of PRRSV. To test this, PAMs transiently transfected with HnRNP F siRNA were challenged with 0.1 multiplicity of infection (MOI) of PRRSV. Cells transfected with a scrambled nontargeting siRNA (NC) were used as control. We analyzed the basal HnRNP F protein expression after transfection of si-HnRNP F in PAMs by real-time PCR and western blotting analysis. Our results showed that HnRNP F mRNA (Fig. 1a) and protein levels (Fig. 1c) were successfully silenced after siRNA transfection. As shown in Fig. 1b, the cell viability assay results suggested that transfection of si-HnRNP F had no obvious cytotoxicity.

We then determined the effect of HnRNP F knockdown on PRRSV replication. As shown in Fig. 1c, silencing of HnRNP F significantly decreased PRRSV RNA expression compared with the NC siRNA transfected group at 24 and 36 hpi. In addition, HnRNP F silencing prominently decreased PRRSV N protein expression levels (Fig. 1d) and PRRSV propagation (Fig. 1e) in PAMs, as detected by western blotting and plaque assay, respectively. These results indicate that silencing of HnRNP F curtails PRRSV infection.

### Ectopic expression of HnRNP F increases PRRSV infection in MARC-145 cells

**Fig. 2:**
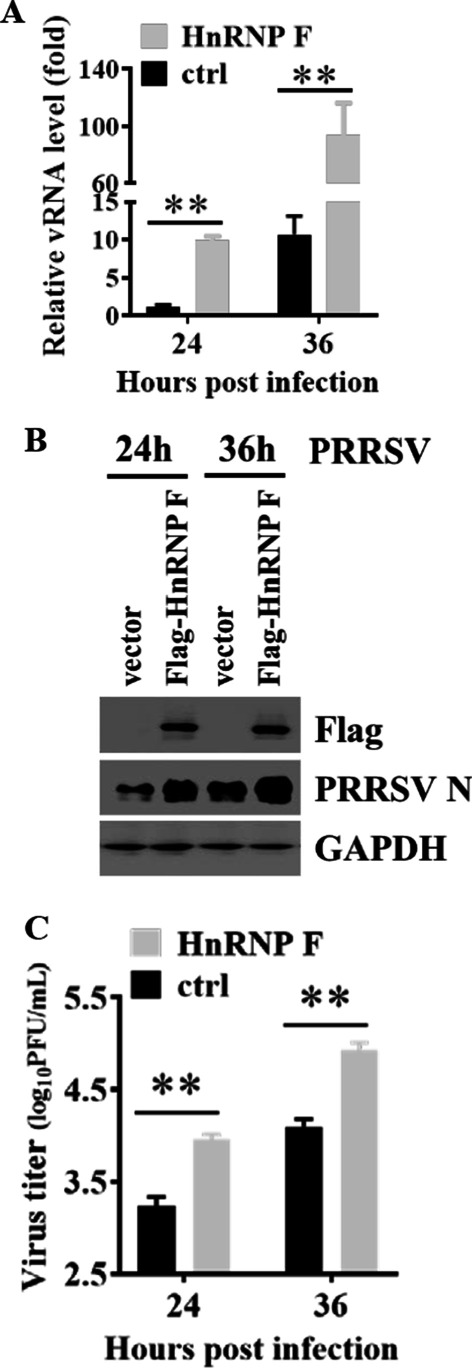
Overexpression of HnRNP F facilitates PRRSV replication. **a**–**c** MARC-145 cells transfected with a control vector or Flag-HnRNP F were infected with 0.1 MOI of PRRSV for 24–36 h. Cells were lysed, and RNA was extracted for real-time PCR analysis of viral RNA (**a**). Expression levels of N protein in cell lysis were determined by western blotting assays (**b**). Culture supernatants containing PRRSV were tittered on MARC-145 cells, and plaques were enumerated (**c**)

To confirm the knockdown results, we further assessed the effect of porcine HnRNP F-overexpression on PRRSV replication in MARC-145 cells. MARC-145 cells were transfected with Flag-HnRNP F plasmids and infected with PRRSV. Transient overexpression of porcine HnRNP F dramatically increased PRRSV total RNA levels in MARC-145 cells compared to the empty vector transfection group (Fig. 2a). According to the western blotting results, MARC-145 cells transfected with Flag-HnRNP F displayed increased levels of viral N protein production compared with the control group (Fig. 2b). Consistently, ectopic expression of HnRNP F markedly increased supernatant virus titers as well (Fig. 2c). These results in Fig. 2 corroborated the data collected in knockdown experiments and strongly support the ability of the porcine HnRNP F to enhance PRRSV propagation.

### HnRNP F selectively binds the GRS

**Fig. 3:**
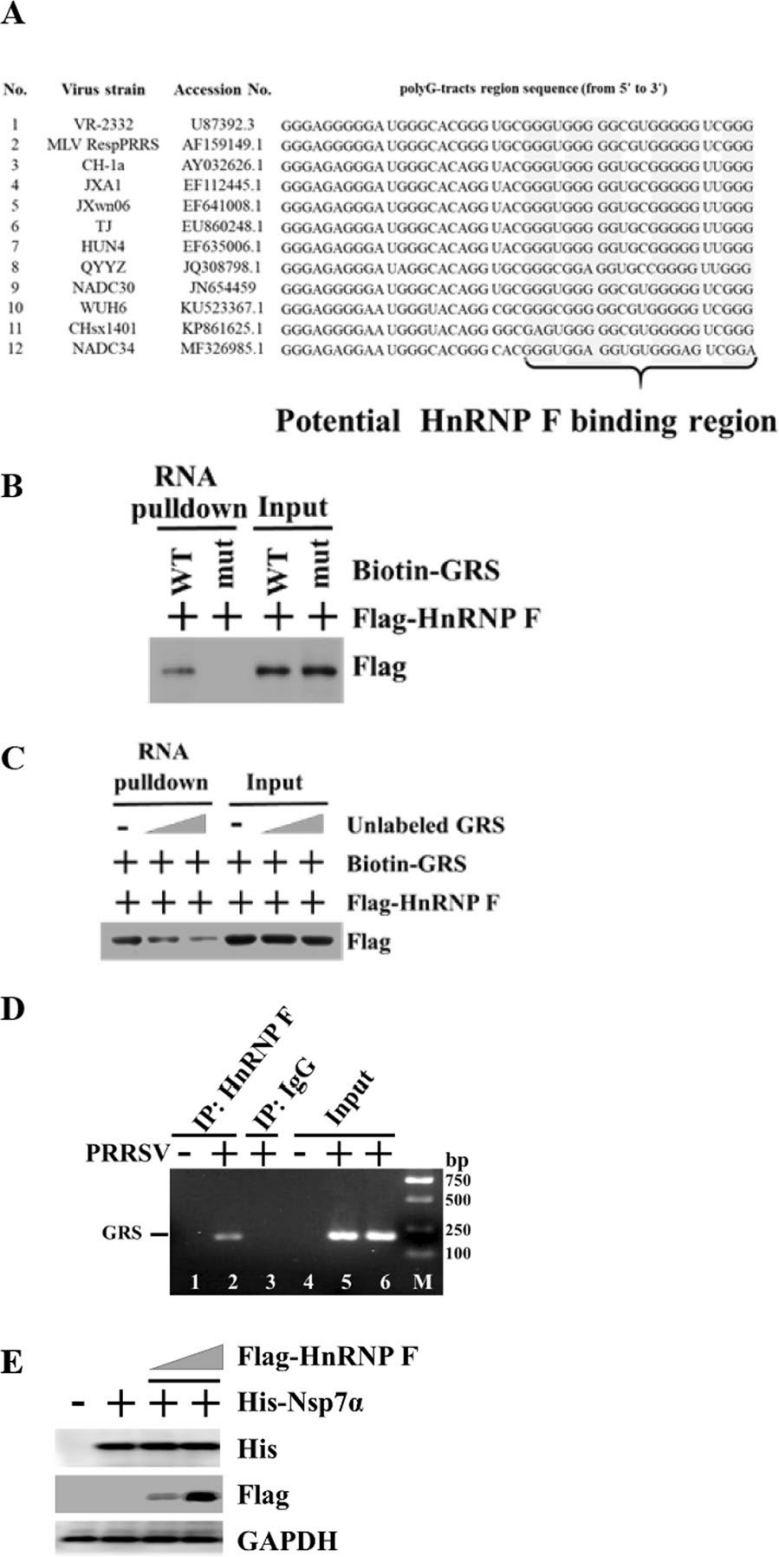
Porcine HnRNP F selectively binds PRRSV GRS. **a** Sequence of PRRSV negative RNA showing putative GRS regions. 10 released PRRSV complete genomes were retrieved from the NCBI website (http://www.ncbi.nlm.nih.gov) and aligned. **b** Wild-type or mutant GRS oligonucleotide was biotinylated and incubated with lysate from HnRNP F transfected HEK293T cells. Streptavidin beads were added to pulldown RNA-bound proteins. The pulldown complexes were eluted and analyzed by western blotting with an anti-Flag antibody. 5% of whole cell extracts were used in reactions as input. **c** HEK293T cells were transfected with Flag-HnRNP F, and cell extracts were incubated with biotinylated GRS along with increasing amounts of unlabeled GRS. Streptavidin beads were added to pulldown RNA-bound proteins, followed by western blotting with anti-Flag antibody. **d** MARC-145 cells were infected with PRRSV (1.0 MOI) for 12 h. The cell lysates were immunoprecipitated with an anti-HnRNP F antibody, or rabbit IgG. RNA was extracted from the immunoprecipitants by protein A + G and subjected to RT-PCR analysis. **e** HEK293T cells seeded in six-well plate were transfected with His-Nsp7α (1 μg), along with Flag-HnRNP F at increasing concentrations (0, 1, and 3 μg). The cell lysates were immunoblotted using anti-His and anti-Flag antibody

Because human HnRNP F has been proposed to regulate splicing by binding GRS sequence motifs embedded in RNA stem-loop structures [18, 19], therefore we speculated that porcine HnRNP F might bind to similar motifs in viral RNAs. Based on pattern matching using the schema GxNyGxNyGxNyGx (N indicates A, C, or U; x ≥ 3; and 7 ≥ y ≥ 1), we found a putative GRS motif in PRRSV negative-strand replication intermediate RNA corresponding to ORF1a using quadruplex-forming G-rich sequences program (QGRS, http://bioinformatics.ramapo.Edu/QGRS/analyze. php analyzer) [27]. The search parameters were: minimal G-Group Size: 3, loop size from 1 to 7. The GRS of PRRSV VR2332 strain contains eight G-tracts motifs comprising of three to five guanines (Fig. 3a). We retrieved ten available genomic sequences and conducted bioinformatics analysis to the level of sequence conservation. Based on genomic variance analysis, PRRSV was classified into two genotypes with varied subtypes. The PRRSV-1 viral RNA poorly aligned with the other sequences and was not added to the alignment. As illustrated in Fig. 3a, this conserved sequence compliments to the coding region for the viral Nsp7α (PRRSV genome region 9181–9414 nt) and possessed five to eight G-tracts within 45 nucleotides which could adopt G4 structures. Therefore, this sequence was selected as target for the subsequent studies.

Next, we determined whether the binding specificities of HnRNP F for viral GRS mirrored those for cellular RNAs. The GRS RNA fragment was synthesized with 1 or 2 additional nucleotides at both ends to ensure they remained in a more natural sequence context. The studied GRS were biotinylated at its 3′ end, thus allowing fragment capture by streptavidin–agarose. A modified single-strand RNA (substituted the middle G to A in each of five G-tracts to disrupt the binding site, termed GRS-mut) incapable of forming a G4 is taken as a negative control. We performed a biotin-conjugated synthetic-GRS plus streptavidin pulldown assay with HEK293T cell lysates, and then subjected this to SDS-PAGE. Western blotting analysis using an anti-Flag antibody showed explicit binding of HnRNP F to the bait WT GRS sequence (Fig. 3b). Nevertheless, biotin-conjugated synthetic GRS-mut could not pulldown HnRNP F (Fig. 3b). These results suggest that the binding of HnRNP F to GRS might depend on the tandem G-tracts.

To confirm that HNRNP F specifically binds to GRS, we subsequently tested whether unlabeled single-stranded GRS RNA could compete with the 3′biotinylated GRS for HnRNP F binding in HnRNP F ectopically expressed HEK293T cells. To this end, increasing amounts of unlabeled were added to lysates of Flag-fusion of HnRNP F plus biotin-labeled GRS, followed by pulldown with streptavidin beads. As concentrations of unlabeled GRS increase, we observed a selective decrease in the levels of pulldown HnRNP F (Fig. 3c), supporting the hypothesis that HnRNP F directly associates with GRS.

To further demonstrate the interaction between HnRNP F and GRS under physiological conditions, we used a RIP strategy. As swine HnRNP F reduces PRRSV replication similarly both in MARC-145 cells and PAMs, MARC-145 cells were selected as the research model for the subsequent experiments to examine the underlying molecular mechanism of anti-PRRSV activity exerted by HnRNP F, unless otherwise indicated. Using the PRRSV-infected MARC-145 cell model, cell lysates collected at 36 hpi were subjected to immunoprecipitation with anti-HnRNP F antibody, and a normal mouse IgG, respectively. The immunocomplexes were extracted and amplified by RT-PCR using primers specific for PRRSV GRS. A cDNA band with the expected size (200 bp) was noted in immunoprecipitants brought down by anti-HnRNP F antibody but not by normal IgG (Fig. 3d). Overall, these findings provided strong evidences that HnRNP F directly interacted with GRS in PRRSV antigenomic RNA under physiological conditions.

To examine whether the binding of HnRNP F to G-tracts in viral Nsp7α region affects Nsp7α production, His-tagged Nsp7α plasmid was co-transfected with Flag-HnRNP F expression plasmid into HEK293T cells. Western blot result suggested that the Nsp7α protein expression remains unchanged after HnRNP F-overexpression (Fig. 3e).

### HnRNP F qRRM3 domain is not necessary for interaction with GRS

**Fig. 4:**
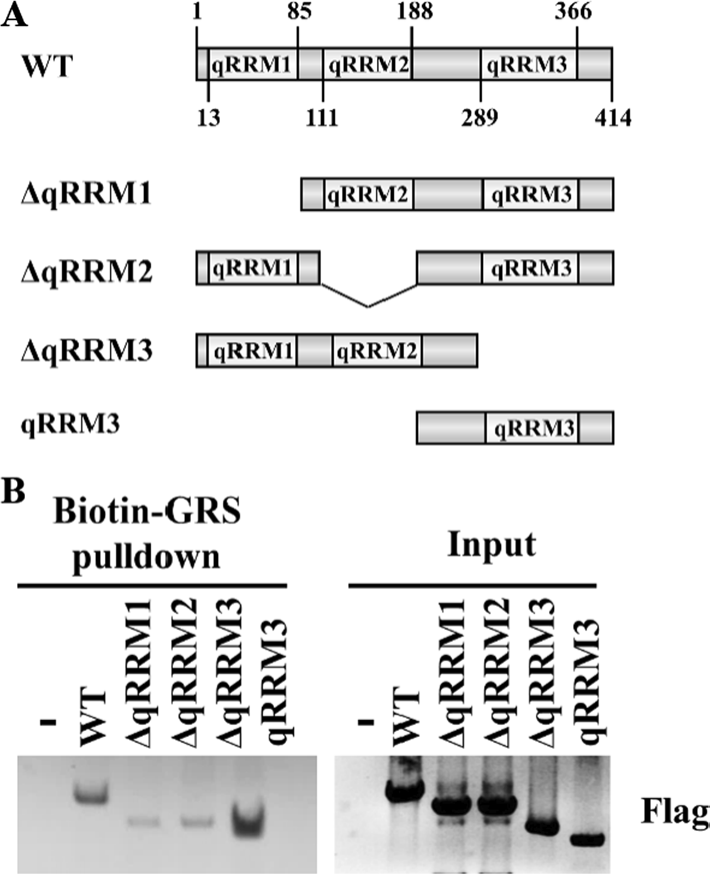
Interaction of GRS with different forms of HnRNP F. **a** Domain architecture of porcine HnRNP F depicting different domains used in this study. **b** GRS oligonucleotide was biotinylated and incubated with lysate from HnRNP F WT, and deletion mutants plasmids transfected HEK293T cells. Then the biotinylated RNA/protein complexes were precipitated by streptavidin beads and analyzed by western blotting

Analysis of the amino acid sequence of HnRNP F has revealed the presence of three qRRMs domains (Fig. 4a), which can recognize G-tracts, maintaining them in a single-stranded conformation co-transcriptionally [19]. To identify the qRRM (s) of porcine HnRNP F involved in binding for GRS, biotin-GRS RNA pulldown experiments were conducted in HEK293T cells transfected with plasmid encoding full-length HnRNP F (WT) and its deletion mutants, followed by pulldown with streptavidin beads. As presented in Fig. 4b, out of the three individual qRRM domain deletion mutants, the ΔqRRM3 mutant consisting of both the qRRM1 and qRRM2 domains appeared the highest affinity for GRS. The mutant containing only the qRRM3 domain failed to bind GRS (Fig. 4b), suggesting that the qRRM3 domain was not required for HnRNP F-GRS interaction. The ΔqRRM1 mutant and the ΔqRRM2 mutant exhibited a significant decreased affinity for GRS compared with the full-length protein (Fig. 4b). Taken together, these results indicate that both the qRRM1 domain and qRRM2 domain of HnRNP F play an important role in directing these binding events.

### PRRSV infection induces the export of HnRNP F from the nucleus to cytoplasm

**Fig. 5:**
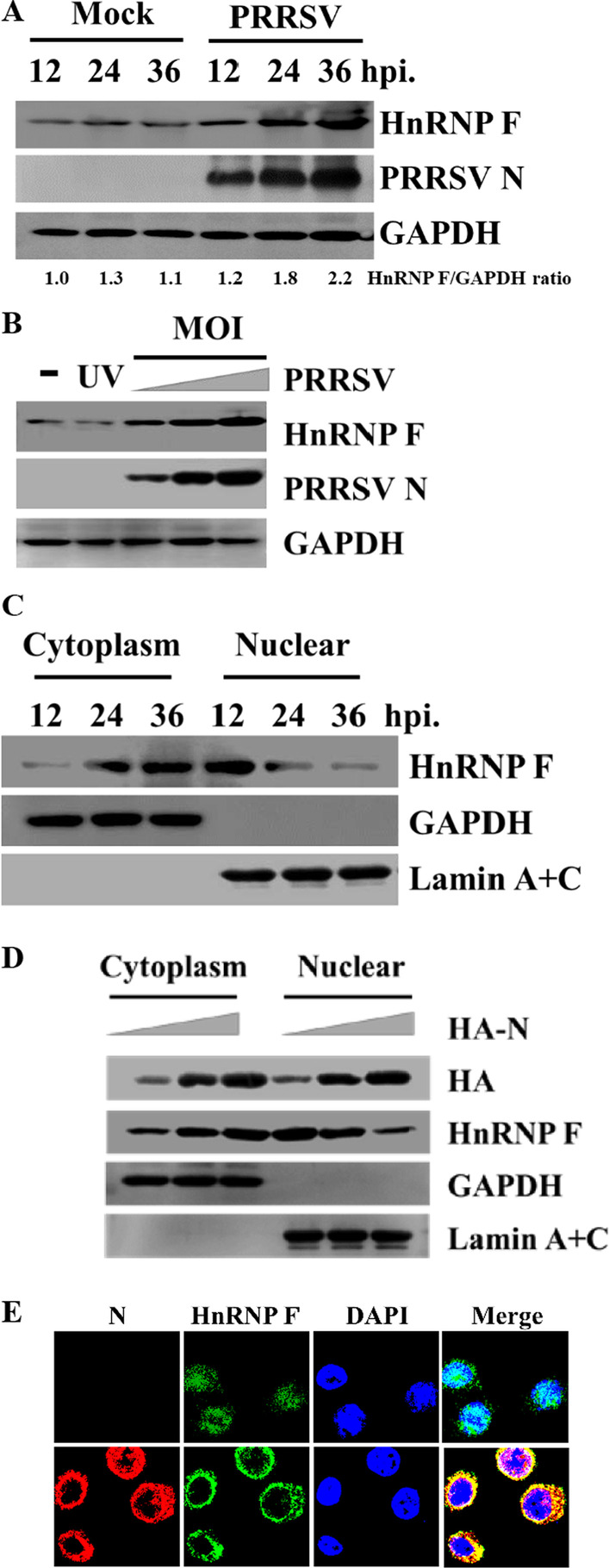
Expression of endogenous HnRNP F in response to PRRSV infection. **a** Western blotting analysis of HnRNP F expression in PAMs infected with PRRSV (MOI = 0.1) at 12–36 hpi. The ratio of HnRNP-F/GAPDH bands was performed using ImageJ software. **b** Western blotting analysis of HnRNP F expression in PAMs infected with different MOI of PRRSV or UV-inactive PRRSV for 36 h. **c** Nuclear/cytoplasmic fractionation of PRRSV-infected PAMs cells, followed by western blotting assessment of HnRNP F expression. The efficacy of the fractionation was analyzed using anti-Lamin A + C antibody, which serves as a nuclear protein marker, and GAPDH, which serves as a cytoplasmic protein marker. **d** Nuclear/cytoplasmic fractionation of pCAGGS-HA-N (1, 2, or 4 μg) transfected MARC-145 cells, followed by western blotting analysis of HnRNP F, GAPDH, and Lamin A + C. **e** PRRSV-infected PAMs were fixed for immunofluorescence assays of HnRNP F (stained with FITC) and N protein (stained with Cy3) using a confocal laser scanning microscope. Nuclei were stained using DAPI (blue)

HnRNPs are abundant nuclear proteins that have distinct signals which enable them to shuttles between the nucleus and the cytoplasm [28, 29], whereas PRRSV replicates mainly in the cytoplasmic compartments. We, therefore, aimed to detect any alteration in the expression of HnRNP F after PRRSV infection. PAMs were infected or mock-infected with 0.5 MOI PRRSV, and protein levels of HnRNP F at various time points were then determined by western blotting. The result shows that PRRSV infection promoted protein expression levels of HnRNP F in PAMs after PRRSV infection, and the level of HnRNP F at 36 hpi was significantly higher than those of other time points (Fig. 5a). To further investigate whether PRRSV infection affects porcine HnRNP F expression, we evaluated the level of endogenous HnRNP F in PAMs infected with increasing doses of PRRSV. Figure 5b shows that the maximal protein level of HnRNP F was expressed at 1.0 MOI, and the induction was dose-dependent.

We then collected the samples at 12–36 hpi for subsequent nuclear and cytoplasmic fraction analysis. As expected, we observed that PRRSV infection results in the re-localization of HnRNP F from the nucleus to the cytoplasm in PAMs (Fig. 5c). In addition, by nuclear and cytoplasmic fractionation and western blotting analysis, we found that overexpression of N protein redistributed HnRNP F from the nucleus into the cytoplasm in a dose-dependent manner, indicating that active viral replication confined these interacting proteins in the cytoplasm (Fig. 5d). Finally, immunofluorescence assays were utilized to visualize the effect of PRRSV infection on the localization of endogenous HnRNP F under physical condition. Result showed that PRRSV infection induced the translocation of HnRNP F from the nuclear to the cytosol (Fig. 5e).

### DHX36 is recruited to N protein-HnRNP F complex upon PRRSV infection

**Fig. 6:**
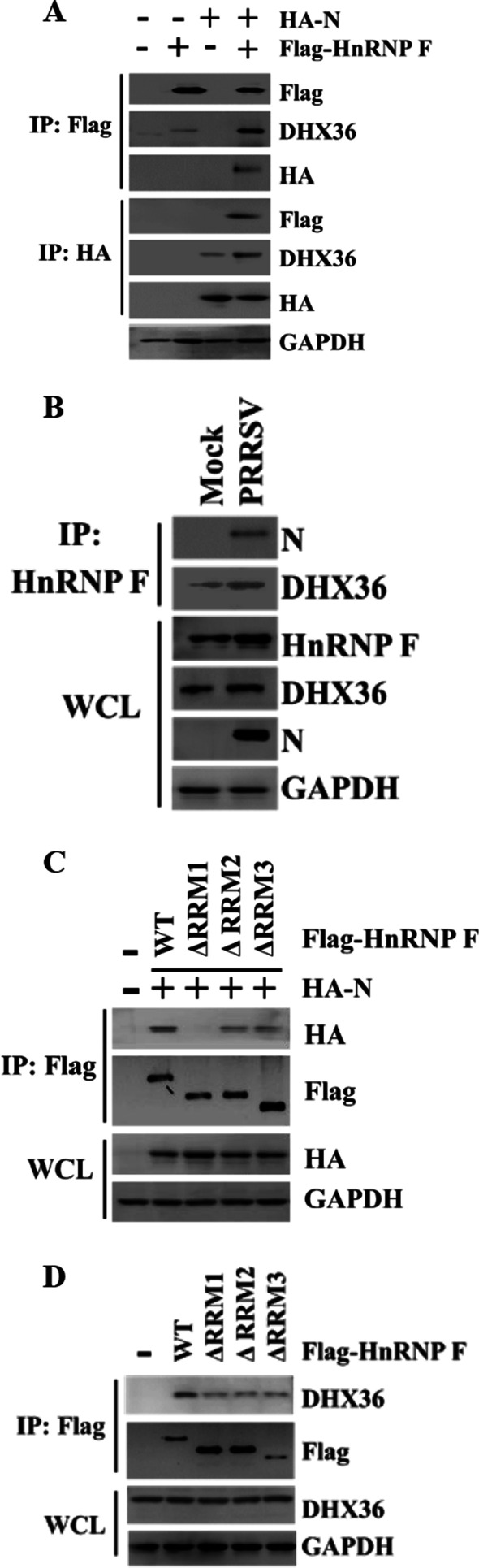
The formation of N–HnRNP F–DHX36 complexes following PRRSV infection. **a** Formation of a putative trimeric complex between PRRSV N protein, HnRNP F, and DHX36 in HEK293T cells transfected with HA-N protein, and Flag-HnRNP F. Lysates were immunoprecipitated and analyzed by western blotting with indicated antibodies. **b** Assessment of the binding of endogenous HnRNP F and DHX36 to PRRSV N protein in PRRSV infected cells using Co-IP. **c** Wild-type HA-N protein was transiently co-expressed with the indicated full-length porcine Flag-HnRNP F or variants in HEK293T cells, precipitated using anti-Flag antibody and co-precipitation of N protein and DHX36 were detected by western blotting. **d** HEK293T cells were transfected with Flag-HnRNP F and its truncation mutants. 36 h later, Co-IP was performed to analyze the interaction between DHX36 and HnRNP F mutants

Many HnRNPs regulate virus infection through association with components of the viral RNA replication complex [30–32]. Mass spectrometry coupled with pulldown assays have found a panel of cellular HnRNP F, associated with the PRRSV N protein, an essential component of the viral RNA replication complex [24, 33]. These findings led us to examine the association of porcine HnRNP F with PRRSV N protein. HA-tagged N protein was co-expressed with Flag-HnRNP F in HEK293T cells. The Co-IP experiments showed that the N protein was efficiently coimmunoprecipitated with porcine HnRNP F (Fig. 6a), suggesting that HnRNP F may be present in the replication complex to function.

Recognition of GRS RNA by HnRNP F is necessary but insufficient for the G4 conformation switch [34]. We speculated that HnRNP F might help revert stable G4 structures via other proteins (i.e., DEAH box helicases) to promote PRRSV RNA synthesis. DHX36, an interacting partner of the PRRSV N protein [35], has been reported previously to cooperate with HnRNP F and regulate the translation of RNA G4-containing DNA damage stress genes [34]. We tested the formation of DHX36-HnRNP F complex in N protein overexpressing HEK93T cells. Flag-tagged HnRNP F was immunoprecipitated from pCAGGS-HA vector or pCAGGS-HA-N protein overexpressing HEK293T cells and analyzed for the presence of DHX36 and N protein. Indeed, Flag-tagged porcine HnRNP F not only co-immunoprecipitated with HA-N protein, but also with DHX36 (Fig. 6a). Interestingly, the amount of DHX36 recruited by Flag-HnRNP F in HA-N protein overexpressing cells was increased when compared to the vector transfected group, suggesting that the recruitment is facilitated by N protein (Fig. 6a). On the other hand, overexpression of Flag-HnRNP F also facilitates dimeric complexes N-DHX36 formation in Co-IP experiments (Fig. 6a).

To investigate whether PRRSV infection influences binding of DHX36 to the N-HnRNP F, MARC-145 cells were infected with PRRSV. The association of DHX36 and N with HnRNP F was detected at 36 hpi. We found that DHX36 binds to HnRNP F in an unstimulated manner, albeit to a lesser extent, compared with the PRRSV-infected group, confirming the interaction of N protein with endogenous DHX36 and HnRNP F (Fig. 6b).

We next investigated the domains of HnRNP F responsible for the association. As shown in Fig. 6c, the construct with qRRM1 domain deletion yielded no signals when detection using an anti-HA antibody. Flag-tagged WT, and two deletion versions of HnRNP F (ΔqRRM2, and ΔqRRM3) were readily immunoprecipitated from lysates of HA-N protein transiently transfected HEK293T cells, suggesting that the interaction between HA-N protein and Flag-HnRNP F is mediated by the qRRM1 domain. Likewise, the DHX36-HnRNP F association was also assessed and the result showed that the interaction between HnRNP F and DHX36 was partially decreased, but not abolished in response to deletion of either one of the qRRM1-3 domains of HnRNP F (Fig. 6d). Thus, the three qRRM domains are all involved in the interaction between DHX36 and HnRNP F.

## Discussion

Upon infection, the PRRSV genome replicates by first synthesizing the negative-sense strand from the positive-sense strand, which is then used as a template to synthesize more positive-sense strands for packaging into new viral capsids. In the current study, our results clearly revealed that HnRNP F selectively associated with GRS elements in the negative-sense strand of PRRSV and identified the formation of G4 helicase DHX36-N protein-HnRNP F complexes upon PRRSV infection. These findings might provide new insight into a plethora of studies on the role of HnRNP proteins in the replication cycle of PRRSV.

In fact, a growing body of evidences suggested that members of the HnRNP family represent a large group of RNA binding proteins that regulate virus reproduction cycle. In prior studies, several viral RNA associated HnRNPs, such as HnRNP D [36] and HnRNP L [37] are required for the translation mediated by hepatitis C virus (HCV) IRES. At the same time, HnRNP A1 [38], HnRNP K [39], and HnRNP L [40] facilitate HCV replication in part by interacting with the virus 5' end genomic RNA. Likewise, HnRNP I and HnRNP C bind to the 3' end of the HCV genome, which are necessary for the initiation and regulation of replication [41]. The involvement of multiple HnRNPs, which act at different stages of the replication cycle through different mechanisms in one single viral infection highlights the importance of HnRNPs. Although HnRNPs were at the center of host-virus cross-talk, a comprehensive understanding of the HnRNPs interacting with the PRRSV replication machinery still remains to be elucidated. Strikingly, the PRRSV replication intermediate RNA contains a conserved tandem G-tracts nucleic acid that could adopt a tetra-stranded structure called G4 [42]. G4 has been initially identified in crucial regulatory regions of the human telomeres, DNA replication origins, and oncogene promoters [20]. As one of the most stable structures, G4 formation in the viral genome has been shown to hinder the promoter activity of HIV [43], human papilloma virus [44], and Herpesvirus [45], as well as inhibits the expression of the GRS harboring genes, as evidenced by the core gene of HCV [46], the L gene of Ebola virus [22], and both the L and G genes of Nipah virus [47], which results in inappropriate viral replication. The first evidence for the sensing of viral G4 by HnRNPs was discovered in HIV LTR, in which HnRNP A2/B1was reported to unwind the stable G4 and enhance transcription [48]. Recently, HnRNP A1 was identified to serve as a G4 unwinding helicase in Kaposi′s sarcoma-associated herpesvirus infection, which regulates viral latency-associated nuclear antigen gene translation [49]. However, whether other HnRNPs exist that can associate with viral GRS to regulate key steps of viral infection remain ambiguous and deserve further investigation.

We found that the interaction of HnRNP F with the GRS in viral negative RNA enhances PRRSV replication. Initially, HnRNP F was found to be generally expressed and involved in a neural-specific pre-mRNA splicing event [50]. Importantly, it showed that HnRNP F bind to the proapoptotic regulator Bcl- 5'-splice site, which contains multiple consecutive G-tracts that have the potential to assemble into G4 [18]. But unlike HnRNP A1, which is preferentially associated with structured G4 [51], HnRNP F might prefer to bind the GRS RNA in its unfolded state to prevent G4 formation [19]. Besides, our data also established HnRNP F as an interaction partner of an additional G4 helicase DHX36. This finding was consistent with previous studies showing that DHX36 prefers to bind structured G4 and act in synergy with HnRNP F/H [34]. HnRNP F could promote the resolution of G4 and maintain G4 in an unfolded conformation by capturing the single-stranded RNA as it is released by G4 helicase DHX36 [19]. Further experiments are needed to characterize the structure and dynamics of how HnRNP F impacts G4 and RNA synthesis during PRRSV infection.

The structural features of viral RNAs often resemble cellular RNAs to effectively utilize cellular RNA-binding proteins to regulate numerous vital steps in replication [49, 52]. The PRRSV conserved 5′ and 3′ untranslated regions (UTR), flanking the positive-sense RNA viral genome, are also characterized by complicated secondary or tertiary structures assembled with stem-loops and pseudoknots, which function as critical contact sites for the specific assembly of the RNA–protein complexes. Due to these factors, PRRSV has strategically evolved to utilize multiple cellular proteins to bind to viral UTR and successfully complete their infection cycle. Among them, cellular poly(C)-binding proteins (also called HnRNP E) [53] and CD151 [54] positively affect PRRSV infection, whereas retinoic acid-inducible gene I (RIG-I) and toll-like receptor 3 (TLR3) recognize PRRSV 3' UTR pseudoknot region and strongly induced type I interferons to curtail viral infection in PAMs [55]. Since current anti-viral drugs and vaccines were compromised due to the emergence of PRRSV variant strains, the distinct features of viral nucleic acids and unique sequence, as well as conserved structural features can be targeted by anti-viral drugs. In this regard, the involvement of G4 structures in several viruses has propelled the development of G4 ligand that directed against viruses [44, 56].

## Conclusion

In summary, these findings provide functional insight into the recognizing of viral conserved GRS RNA by porcine HnRNP F that favors PRRSV propagation, as well as an alternative approach for the development of specific anti-viral strategies and genomic engineering-based vaccines.

## Data Availability

All data generated or analyzed during this study are included in the article.

## Abbreviations

PRRSV: Porcine reproductive and respiratory syndrome virus
HnRNP F: Heterogeneous nuclear ribonucleoprotein F
G-tracts: Guanine tracts
GRS: Guanine-rich segments
N: Nucleocapsid
G4: Guanine-quadruplex
ORF: Open reading frames
Nsps: Non-structural proteins
qRRM: Quasi-RNA recognition motifs
PAMs: Porcine alveolar macrophages
RIPA: Immunoprecipitation lysis buffer
Co-IP: Coimmunoprecipitation assays
HEK293T: Human embryonic kidney 293T
PFU: Plaque-forming unit
RIP: RNA immunoprecipitation
MOI: Multiplicity of infection
hpi: Hours post infection
HCV: Hepatitis C virus
UTR: Untranslated regions
